# Supplementary material for: In situ structure and dynamics of an alphacoronavirus spike protein by cryo-ET and cryo-EM
Source: Nat Commun. 2022 Aug 19;13:4877. doi: 10.1038/s41467-022-32588-3 (PMC9388967; doi:10.1038/s41467-022-32588-3)
Supplement: Supplementary file 1 — Supplementary Information [file 41467_2022_32588_MOESM1_ESM.pdf]

Supporting information

In situ structure and dynamics of an alphacoronavirus spike protein by cryo-ET and cryo-EM

Cheng-Yu Huang[1,#], Piotr Draczkowski[1,2,#], Yong-Sheng Wang[1,3,#], Chia-Yu Chang[1,4,#], Yu-Chun Chien[1,3], Yun-Han Cheng[4], Yi-Min Wu[5], Chun-Hsiung Wang[5], Yuan-Chih Chang[5], Yen-Chen Chang[4], Tzu-Jing Yang[1,3], Yu-Xi Tsai[1,3], Kay-Hooi Khoo[1,3], Hui-Wen Chang[4], and Shang-Te Danny Hsu[1,3,*]

1. Institute of Biological Chemistry, Academia Sinica, Taipei 11529, Taiwan
2. Faculty of Pharmacy, Medical University of Lublin, ul. W. Chodzki 4a, 20-093 Lublin, Poland
3. Institute of Biochemical Sciences, National Taiwan University, Taipei 11529, Taiwan
4. Graduate Institute of Molecular and Comparative Pathobiology, School of Veterinary Medicine, National Taiwan University, Taipei 10617, Taiwan
5. Academia Sinica Cryo-EM Center, Academia Sinica, Taipei 11529, Taiwan

* Corresponding author: sthsu@gate.sinica.edu.tw

# These authors contribute equally

Contents:

Supplementary Figures 1-17

Supplementary Tables 1-6

Supplementary Movies 1-2 are provided separately as .mp4 files

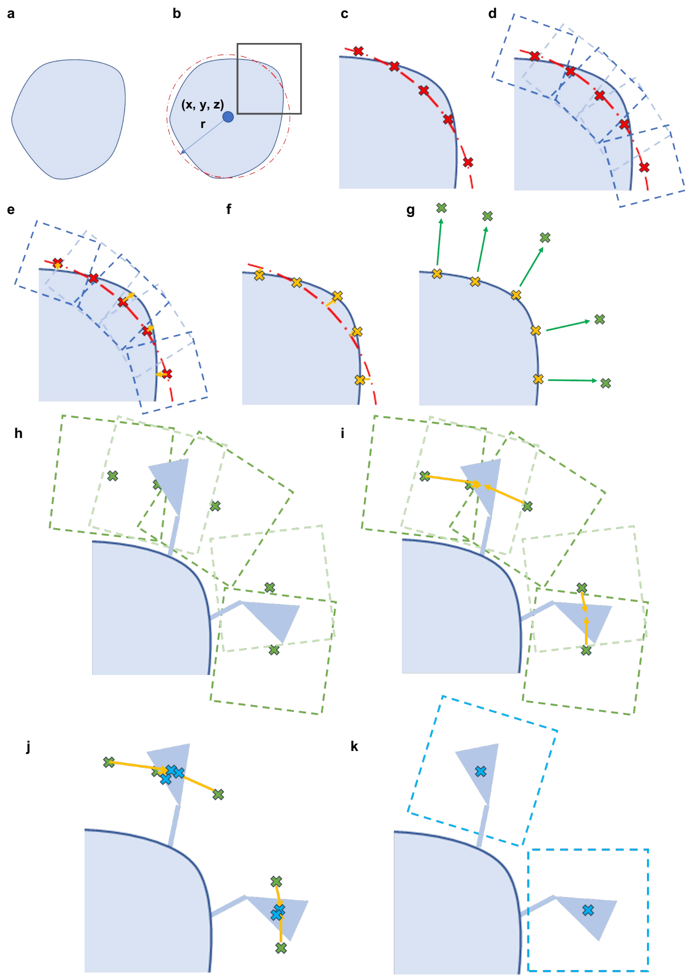

Supplementary Fig. 1. Schematic illustration of the subtomogram analysis for the PEDV S. (a) Schematic illustration of a virus in the cryo-ET sample. **(b)** The center (x, y, z) and the radius (r) of the virus were manually measured. **(c)** An over-sampling grid of coordinates as indicated by red crosses was generated on the surface of the sphere defined. **(d)** Subtomograms boxed by dashed blue squares were extracted at the coordinates. **(e)** Subtomogram averaging was performed, and the subtomogram coordinates were shifted towards the true virus membrane subsequently **(f)** The new coordinates indicated by yellow crosses defined the virus membrane curvature. **(g)** Subtomogram coordinates were shifted away from the membrane for 14 nm where we expect the S trimers should locate as indicated by green crosses. **(h)** The subtomograms boxed by dashed green squares were re-extracted. **(i)** Subtomogram averaging was performed again, but this time using the low-resolution template structure of the S trimer that was generated previously as a template. The subtomogram coordinates were shifted towards true locations of PEDV S as indicated by yellow arrows. **(j)** A cluster of subtomogram coordinates formed around the true location of PEDV S as a result of coordinates shifting. **(k)** Distance clean was performed, and only one subtomogram coordinate remains in each cluster.

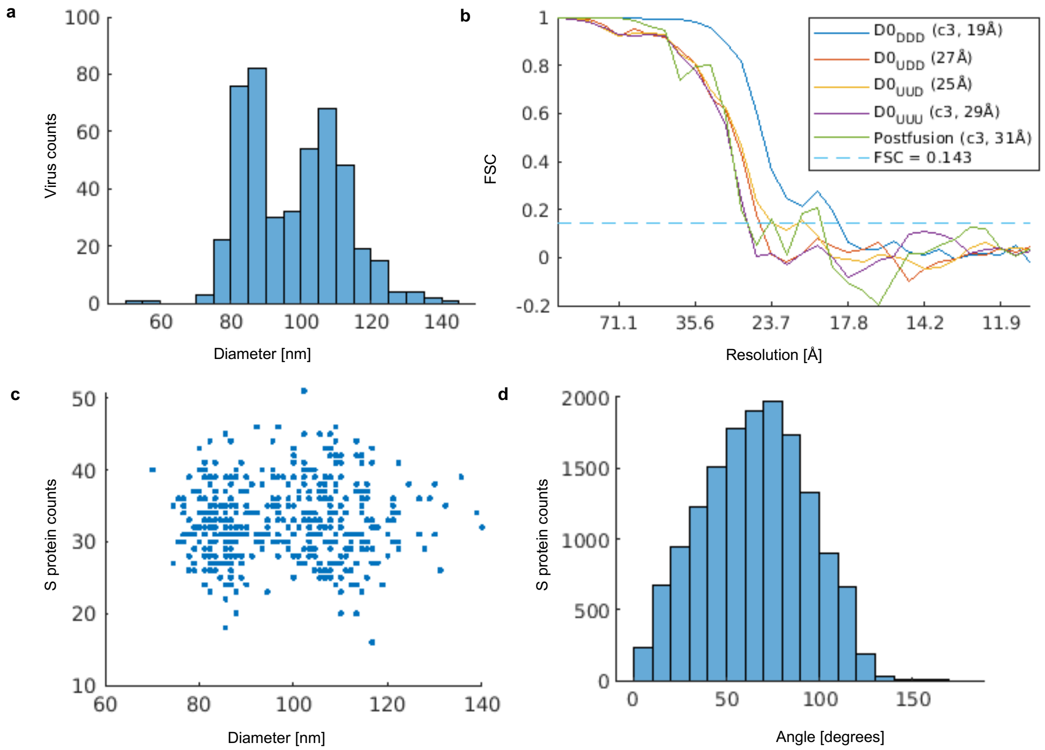

Supplementary Fig. 2. Geometrical analysis of viral particles and cryo-ET resolution analysis. (a) Histogram of the viral particle diameters, which shows a bimodal distribution. **(b)** Fourier shell correlation (FSC) of the prefusion PEDV S structures with four different D0 arrangements and postfusion PEDV S structure. **(c)** Scatter plot of identified PEDV S counts as a function of viral particle diameter. **(d)** Histogram of the tilt angle with respect to the normal of the membrane.

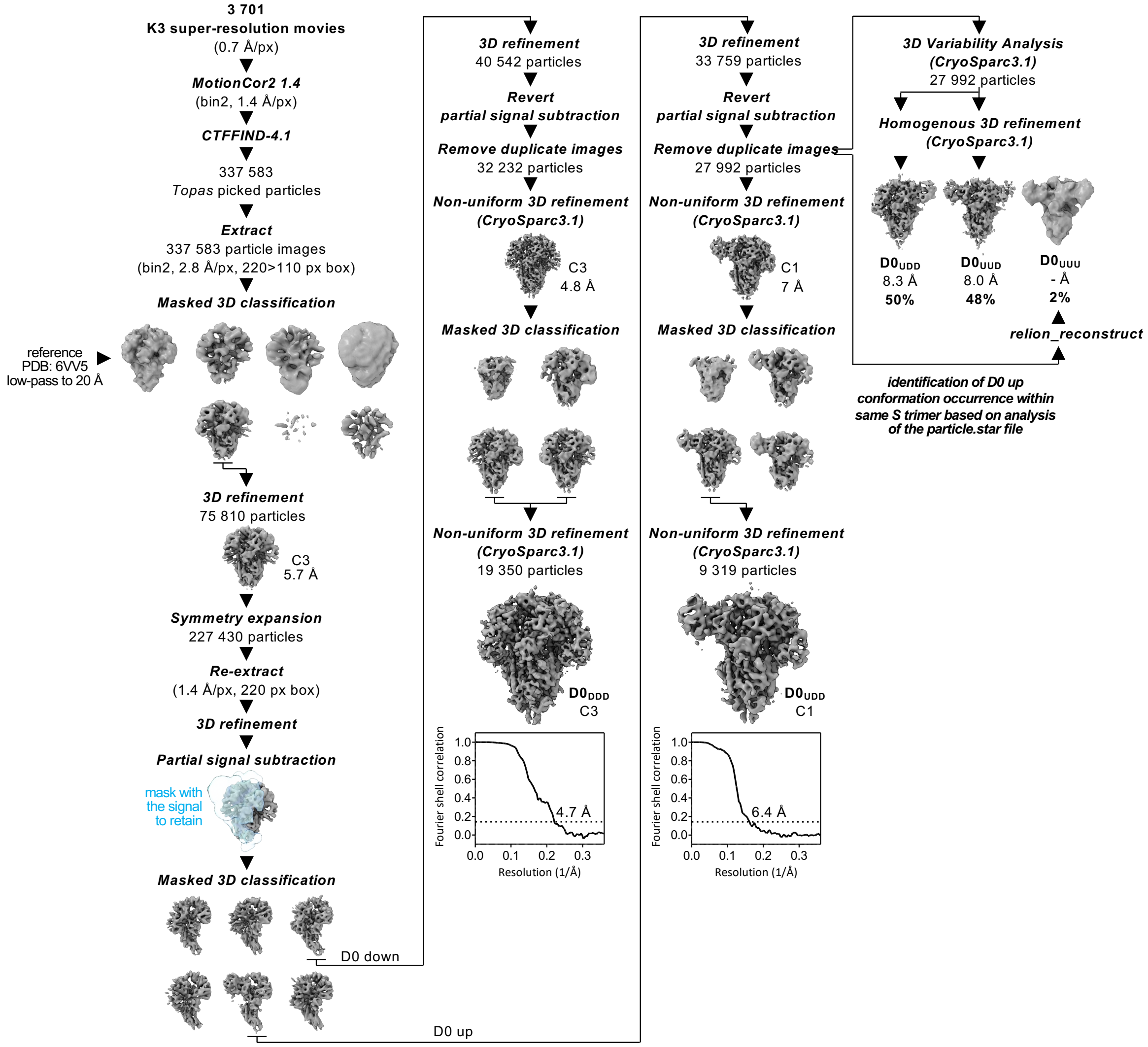

Supplementary Fig. 3. Overview of cryo-EM data processing pipeline of intact virus-derived PEDV PT52 S.

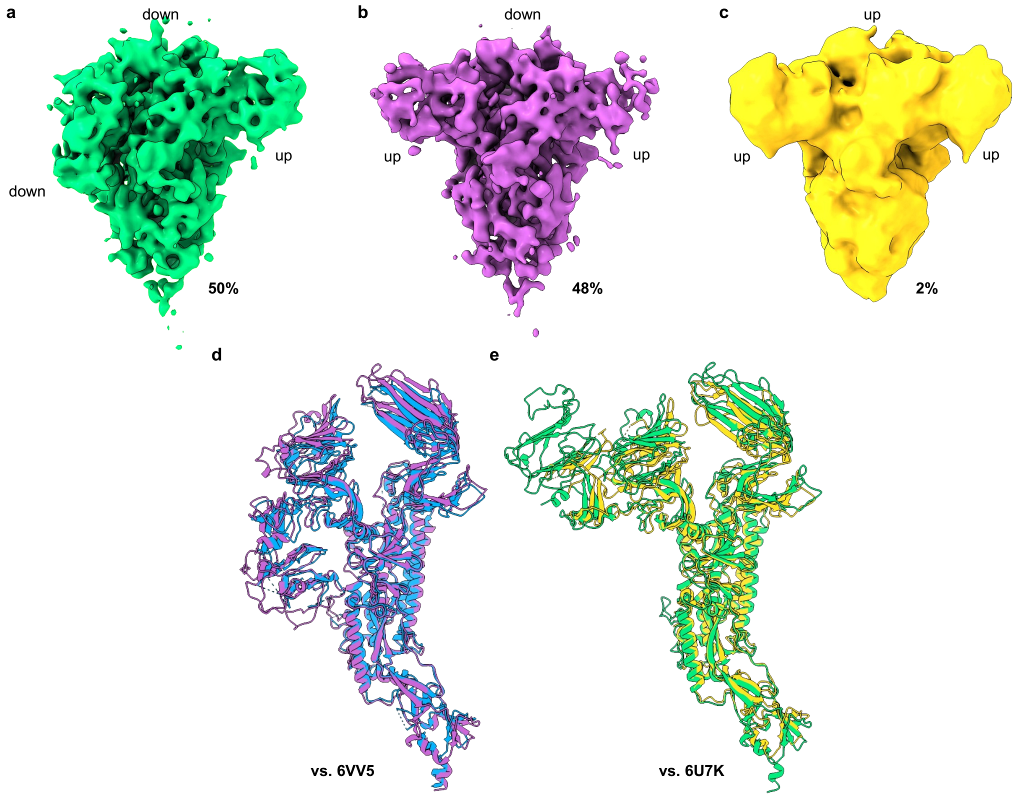

Supplementary Fig. 4. Heterogeneity of the PEDV S revealed by cryo-EM. The maps of the alternative conformations of the PEDV PT52 S trimer incorporating one **(a)**, two **(b)** or all three protomers **(c)** in the D0-up state. Comparison of the refined protomer models in the D0-down **(d)** and -up **(e)** states with the previously reported structures of PEDV S variants.

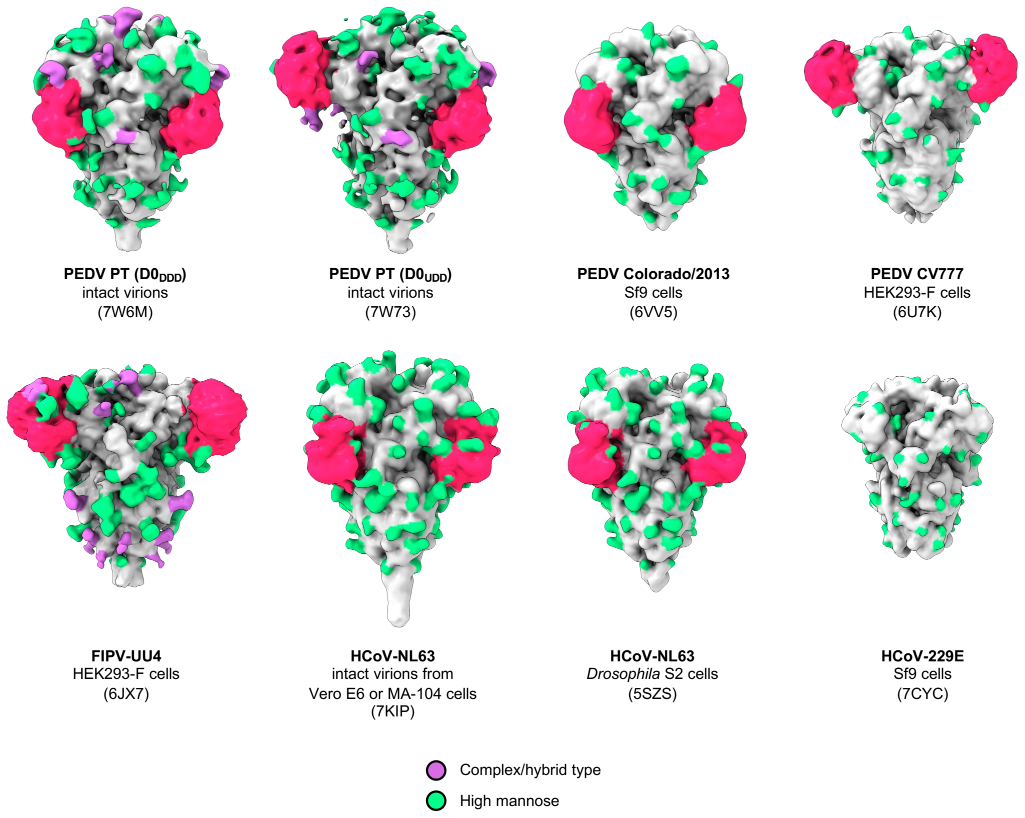

Supplementary Fig. 5. Comparison of the N-glycosylation patterns and the D0 conformations of reported alphacoronavirus S protein structures. All the maps were low-pass filtered to 10 Å to facilitate identification of the glycan densities (green and purple). The D0 in individual structures are colored pink.

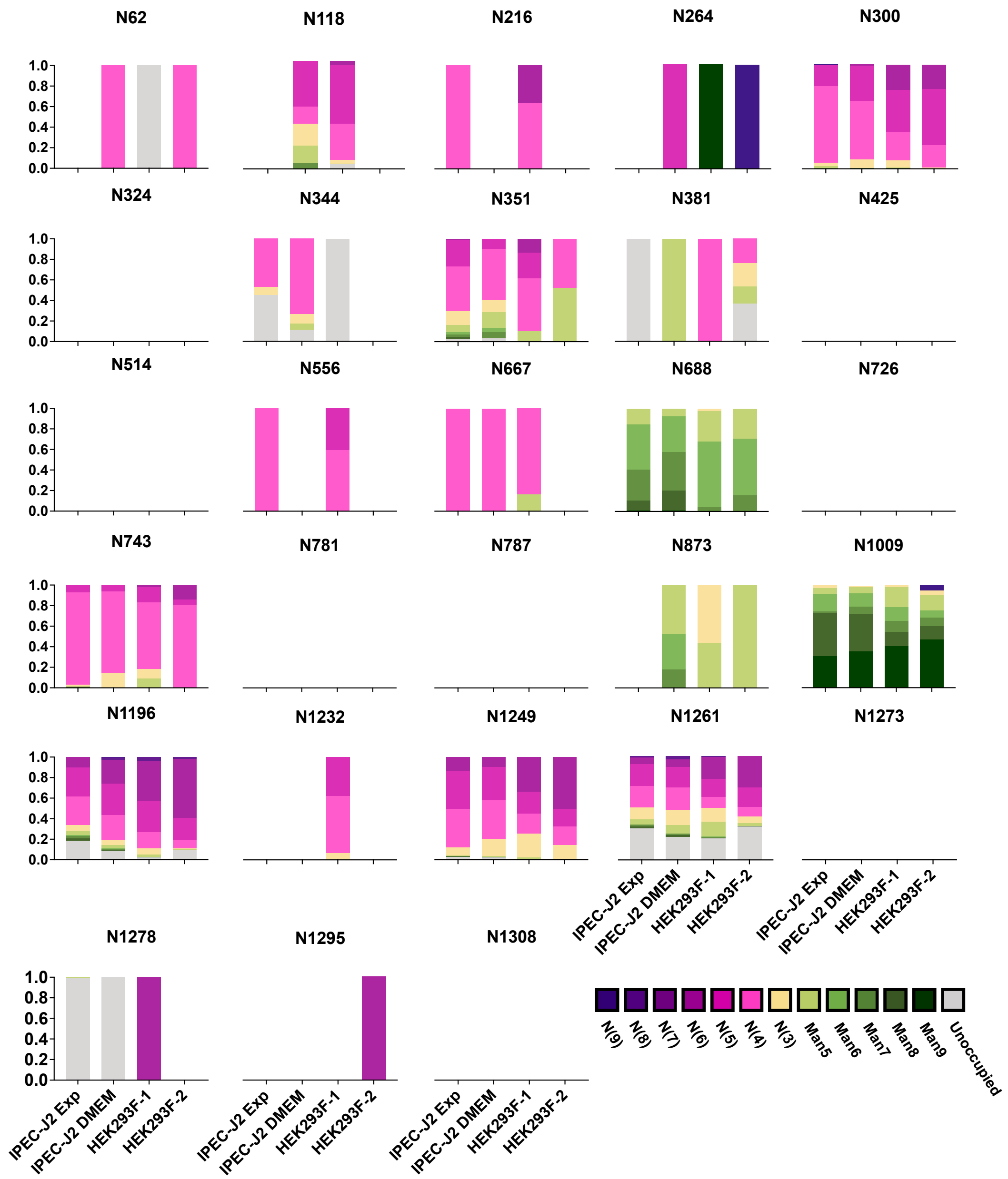

Supplementary Fig. 6. Quantitative MS glycopeptide analysis of PEDV PT52 S variants. Normalized distributions of different glycoforms for individual glycosylation sites are shown in stacked bar charts with color coding indicated on the lower right. The IPEC-J2-derived PEDV PT52 S was expressed in two different media, and the HEK293F-derived PEDV PT52 S were analyzed in biological duplicates. The corresponding results are indicated below each stacked bar.

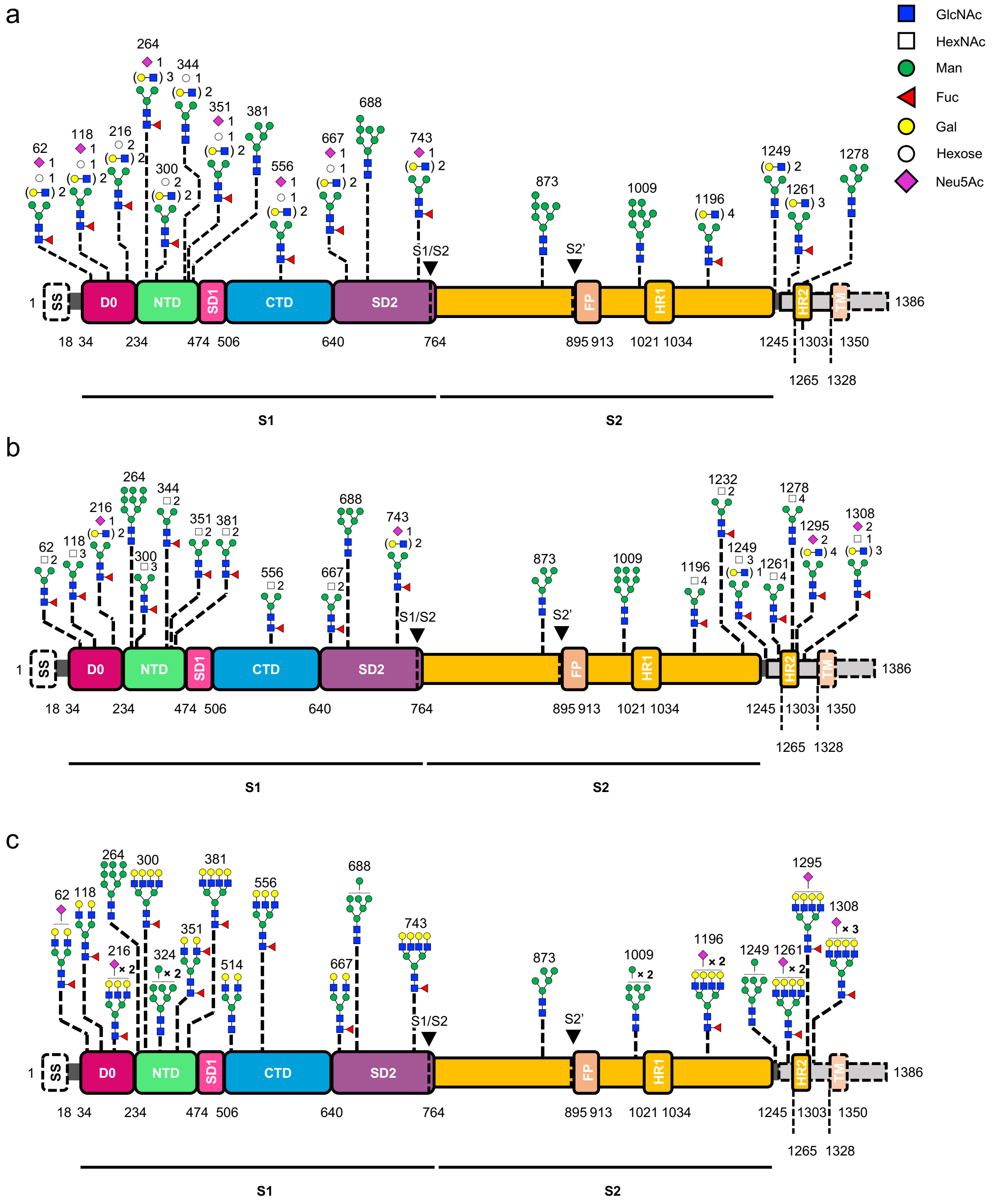

Supplementary Fig. 7. Comparison of representative glycoforms of PEDV S variants. The representative glycoforms of PEDV PT52 S expressed in IPEC-J2 cells **(a)**, HEK293F **(b)**, and PEDV S protein (HC070225) expressed in HEK293 cells **(c)** are shown fin the Symbol Nomenclature For Glycans (SNFG) as indicated on the right. Note that the IPEC-J2 specific Galα1-3Gal linkages, corresponding to the Hexose (open circle) to the Neu5Ac (magenta diamond) linkage, are observed in Asn62, Asn118, Asn216, Asn300, Asn344, Asn351, Asn556 and Asn667.

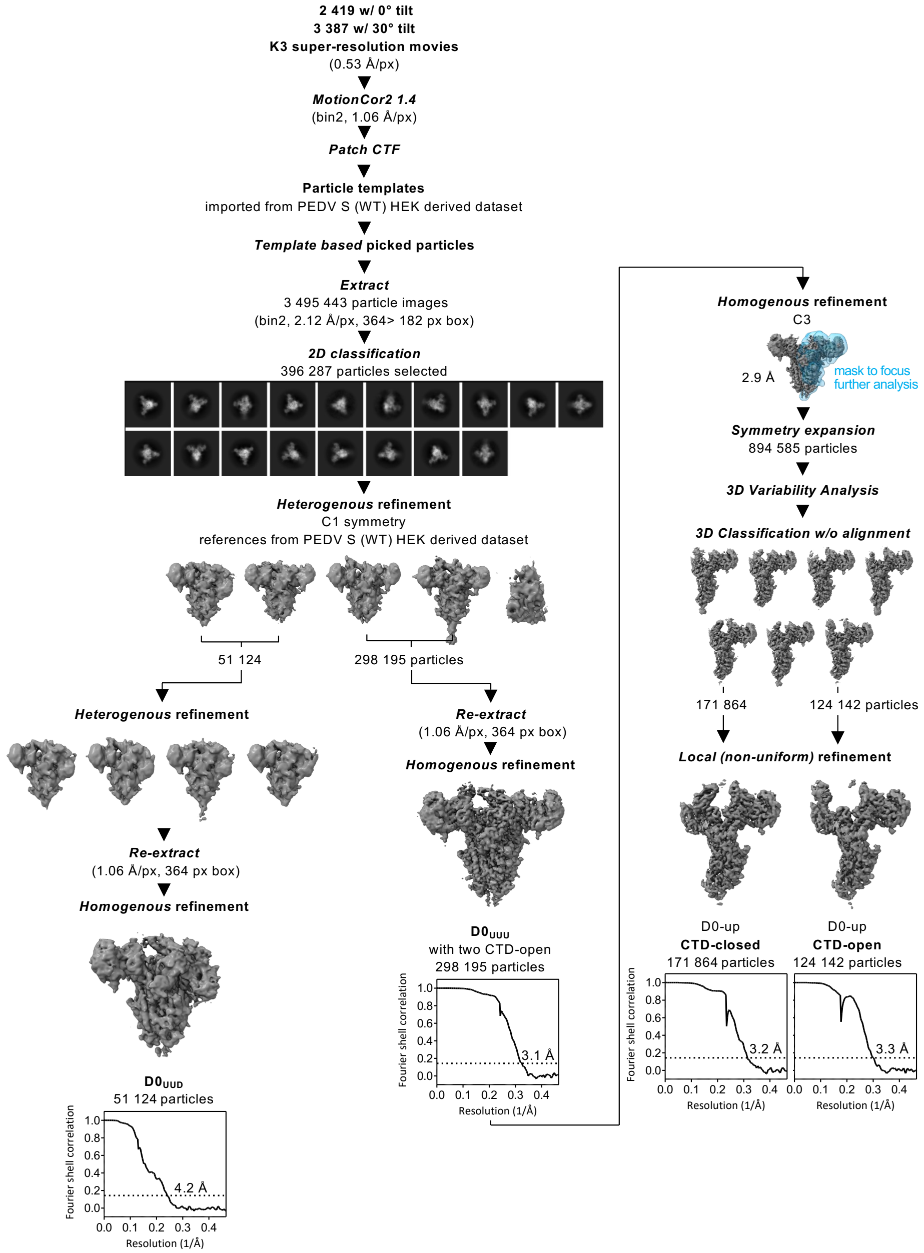

Supplementary Fig. 8. Overview of cryo-EM data processing pipeline of IPEC-J2 cell-derived recombinant PEDV PT52 S.

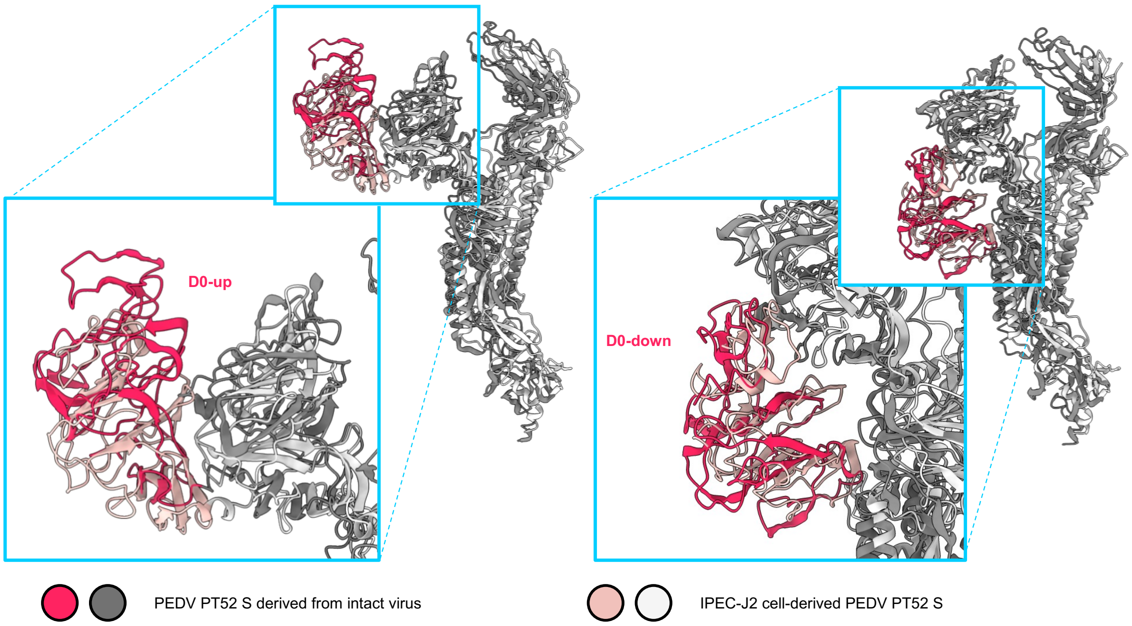

Supplementary Fig. 9. Superimposition of PEDV PT52 S structures derived from intact virus and IPEC-J2 cells. The single protomer structure of PEDV PT52 S with two distinct D0 conformations (up versus down) derived from both intact virus and IPEC-J2 cells were superimposed. The structure of PEDV PT52 S derived from the intact virus was shown in dark gray with D0 colored in deep pink while the one from IPEC-J2 cells was shown in light gray with D0 colored in pink.

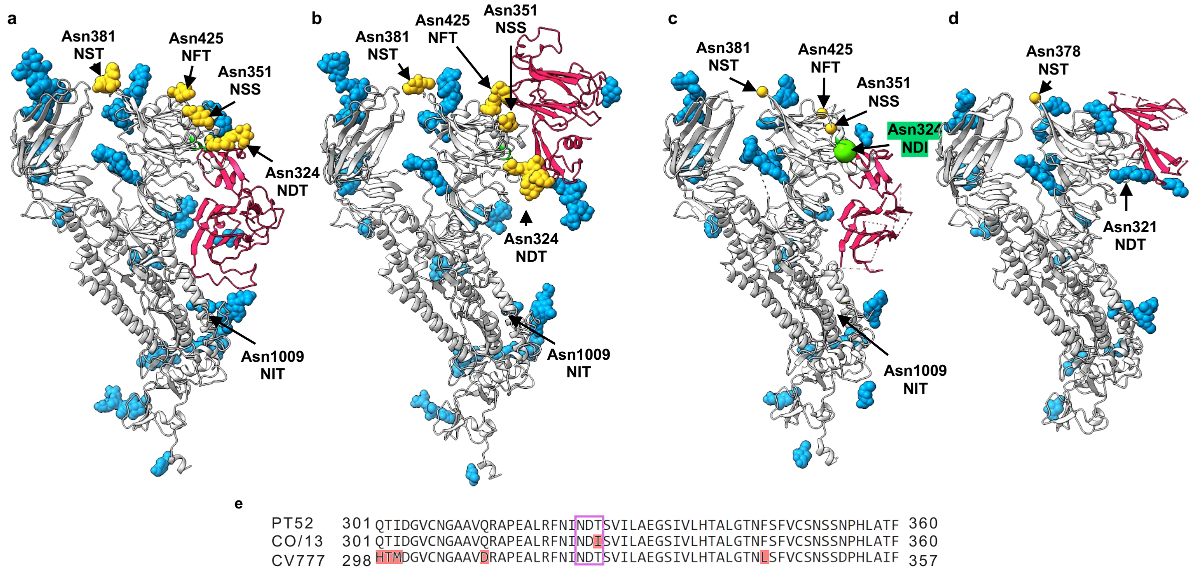

Supplementary Fig. 10. Structural mapping of difference in the N-glycosylation between different PEDV S variants. Glycans observed on the PEDV PT52 S, both the D0-down **(a)** and D0-up **(b)** shown in pink, but absent on the reported models of the S protein from the PEDV CO/13 **(c)** and CV777 **(d)** are highlighted in yellow with their glycosylation sequons displayed. The corresponding unoccupied glycosylation sites on PEDV CO/13 and CV777 S are marked with yellow spheres. The loss of the glycosylation on Asn324 in CO/13 is highlighted in green.

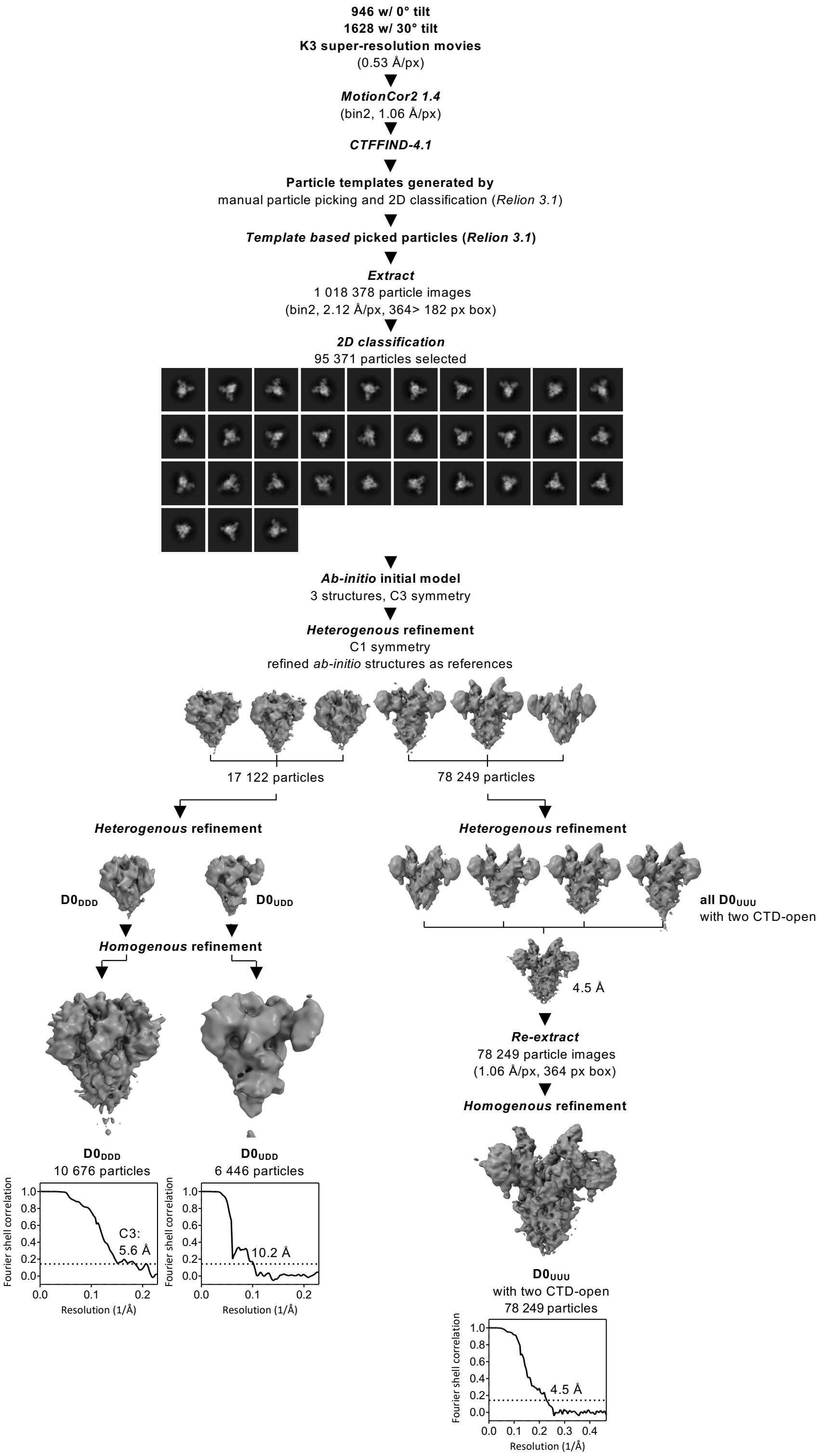

Supplementary Fig. 11. Cryo-EM data processing pipeline of HEK293F cell-derived recombinant PEDV PT52 S.

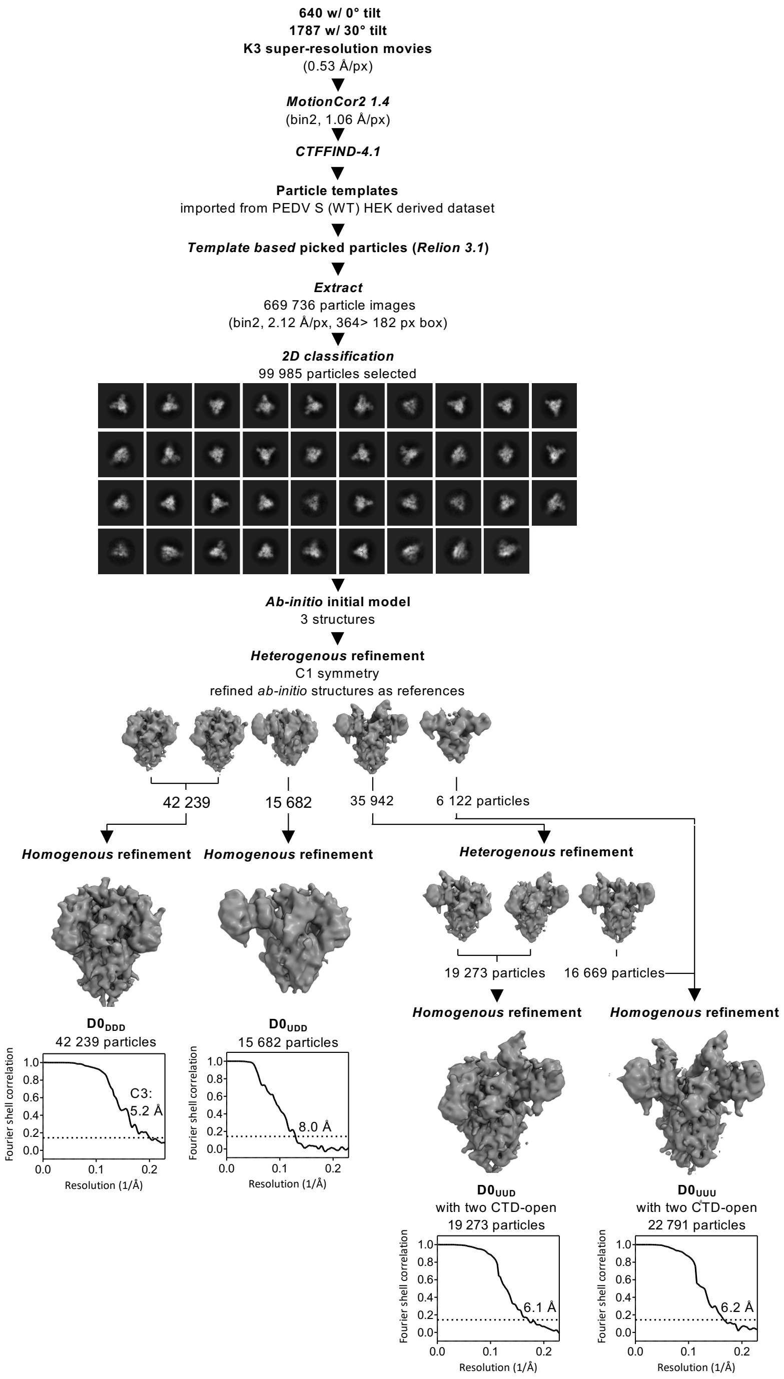

Supplementary Fig. 12. Cryo-EM data processing pipeline of HEK293F cell-derived recombinant PEDV PT52 S T326I.

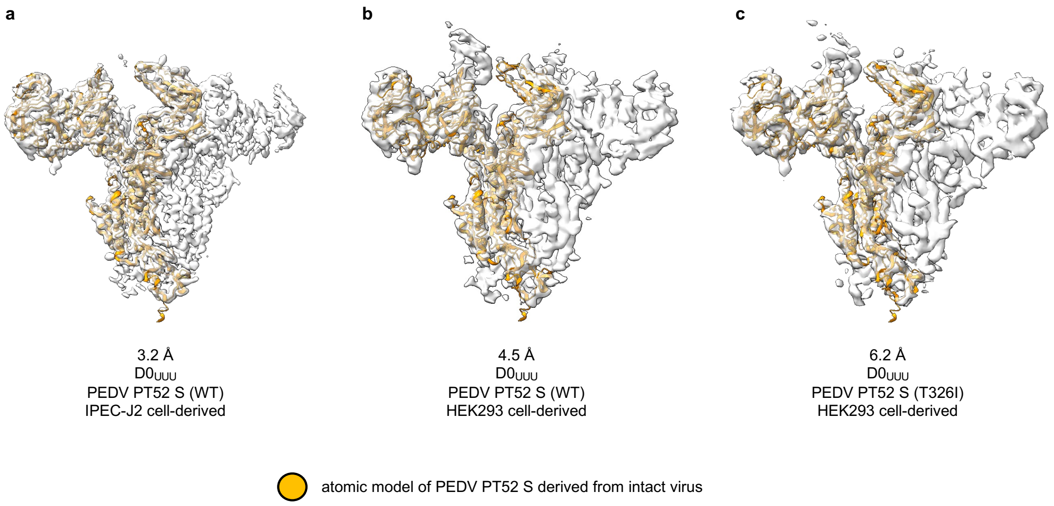

Supplementary Fig. 13. Comparison of cryo-EM maps of recombinant PEDV PT52 S derived from different expression hosts. The cryo-EM maps of PEDV PT52S expressed in IPEC-J2 **(a)** and HEK293 **(b)** cells, and that the T326I variant expressed in HEK293 cells **(c)** are shown in semi-transparent surfaces. The atomic structure of IPEC-J2 cell-derived PEDV PT52 S (shown in orange cartoon representation) nicely fits into the cryo-EM map of HEK293F cell-derived PEDV PT52 S without **(b)** and with **(c)** the T326I mutation without further alterations.

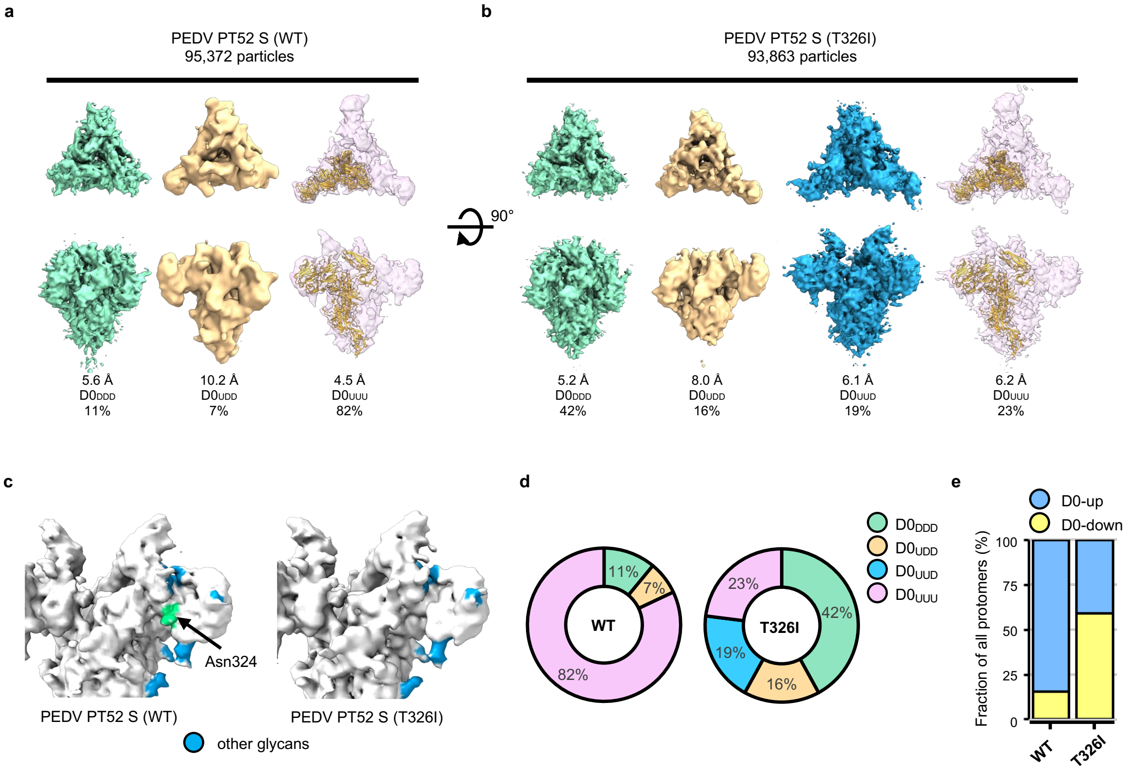

Supplementary Fig. 14. The conformation heterogeneity of recombinant PEDV PT52 S. (a) Three classes of conformation derived from WT were revealed from the cryo-EM data, which corresponded to the $D0_{UUU}$, $D0_{UDD}$ and $D0_{DDD}$ arrangements with 82, 7, and 11 % of the total population, respectively. **(b)**Four classes of conformation derived from T326I were revealed from the cryo-EM data, which corresponded to the $D0_{UUU}$, $D0_{UUD}$, $D0_{UDD}$, and $D0_{DDD}$ arrangements with 23, 19, 16, and 42 % of the total population, respectively. **(c)** The additional density of cryo-EM maps in WT was observed near the Asn324 but absent in T326I. **(d)** The relative ratio of conformation heterogeneity of recombinant PEDV PT52 S with or without T326I. **(e)** Quantification of the relative D0-up vs D0-down conformations at the level of individual protomers.

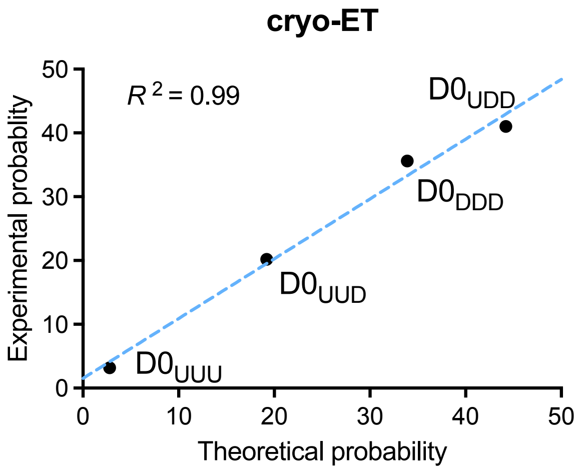

Supplementary Fig. 15. Correlation between the experimental and theoretical populations of PEDV PT52 S in different protomer arrangements. The D0-up probabilities derived from cryo-ET and theoretical populations derived from the single variable of the D0-up probability according to the result from the total cryo-ET subtomograms are shown along the Y- and X-axis, respectively. No interactions between neighboring protomers in each individual S trimers were assumed.

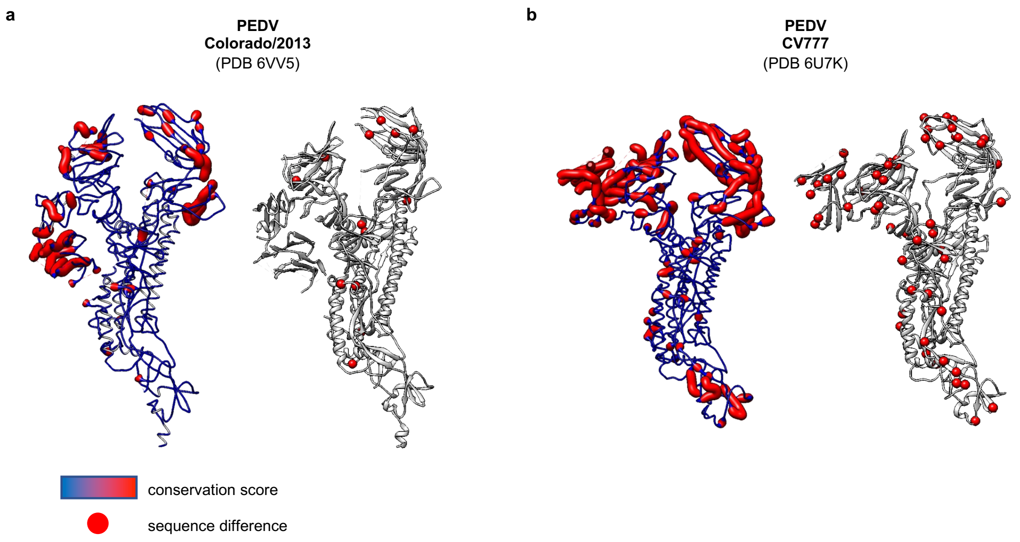

Supplementary Fig. 16. Structure-based sequence alignments of PEDV S variants. The spike protomer of the Colorado/2013 (CO/13) strain **(a)** and the CV777 strain **(b)** are shown in sausage representations (left) and color-ramped from blue to red corresponding to high to low conservation score as indicated by the color scale bar bellow. Regions that exhibit lower sequence conservation are shown in larger radii. The same structures are shown in white cartoon representations (right) with the $C\alpha$ atoms of the residues that differ from the sequence of PEDV PT52 S shown in red spheres.

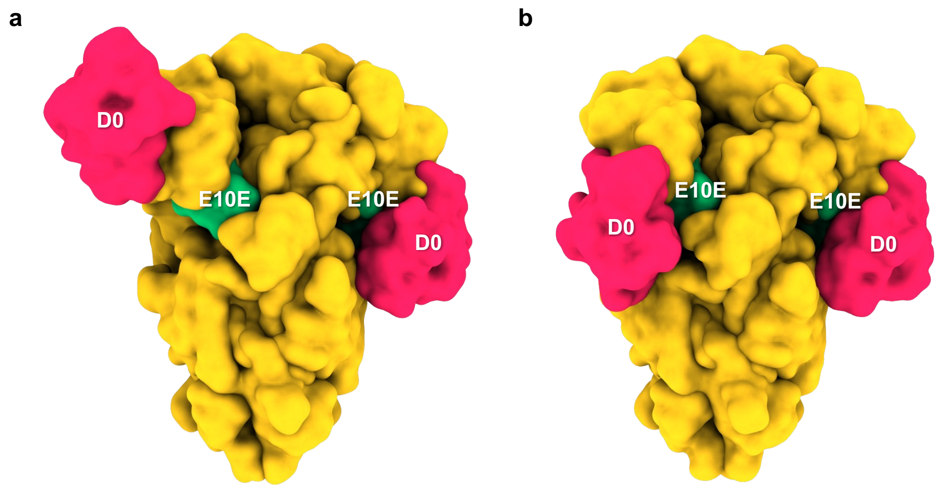

Supplementary Fig. 17. Structural mapping of reported epitopes on the PEDV S protein. The PEDV PT52 S protein structure in the $D0_{UDD}$ (a) and $D0_{DDD}$ (b) configurations are shown in surface representations. The regions corresponding to the D0 and the reported novel epitopes E10E are colored in pink and green respectively. The exposure of the E10E epitope is modulated by the up/down conformation of the D0.

Supplementary Table 1. MS glycopeptide analysis results

Construct			PEDV PT52 S					PEDV HC070225 S
Host cell line			iPECJ2	iPECJ2	HEK293F			HEK293F
Expression medium			FreeStyle™ 293 Expression Medium	DMEM	FreeStyle™ 293 Expression Medium			FreeStyle™ 293 Expression Medium
No.	Nsite	sequon				Nsite	sequon	
1[a]	62	NST		HexNAc(4)Hex(6)Fuc(1)NeuAc(1)	HexNAc(4)Hex(3)Fuc(1)[b]	61	NSS	HexNAc(4)Hex(5)Fuc(1)NeuAc(1)
2[a]	118	NAT	HexNAc(4)Hex(6)Fuc(1)NeuAc(1)[b]	HexNAc(5)Hex(5)Fuc(1)	HexNAc(5)Hex(3)Fuc(1)	131	NKT	HexNAc(4)Hex(5)Fuc(1)
3	216	NVT	HexNAc(4)Hex(7)Fuc(1)	HexNAc(4)Hex(7)Fuc(1)[b]	HexNAc(4)Hex(5)Fuc(1)NeuAc(1)[b]	217	NVT	HexNAc(5)Hex(6)Fuc(1)NeuAc(2)
4	264	NDS	HexNAc(2)Hex(9)[b]	HexNAc(5)Hex(6)Fuc(1)NeuAc(1)[c]	HexNAc(2)Hex(9)	265	NDS	HexNAc(2)Hex(9)
5	300	NQT	HexNAc(4)Hex(7)Fuc(1)	HexNAc(4)Hex(7)Fuc(1)	HexNAc(5)Hex(3)Fuc(1)	301	NQT	HexNAc(6)Hex(7)Fuc(1)
6	324	NDT				325	NDT	HexNAc(2)Hex(8)
7[a]	344	NFS	HexNAc(4)Hex(6)	HexNAc(4)Hex(6)	HexNAc(4)Hex(3)Fuc(1)[b]	345	NLS	
8	351	NSS	HexNAc(4)Hex(6)Fuc(1)NeuAc(1)	HexNAc(4)Hex(7)Fuc(1)	HexNAc(4)Hex(3)Fuc(1)	352	NSS	HexNAc(4)Hex(5)Fuc(2)[b]
9	381	NST	HexNAc(4)Hex(7)Fuc(1)[b]	HexNAc(2)Hex(5)	HexNAc(4)Hex(3)Fuc(1)	382	NST	HexNAc(6)Hex(7)Fuc(1)
10	425	NFT				426	NFT	
11	514	NIT				515	NIT	HexNAc(4)Hex(5)[c]
12	556	NVT	HexNAc(4)Hex(7)Fuc(1)	HexNAc(4)Hex(6)Fuc(1)NeuAc(1)[b]	HexNAc(4)Hex(3)Fuc(1)[c]	557	NVT	HexNAc(5)Hex(6)Fuc(1)[b]
13	667	NSS	HexNAc(4)Hex(6)Fuc(1)NeuAc(1)	HexNAc(4)Hex(6)Fuc(1)NeuAc(1)	HexNAc(4)Hex(3)Fuc(1)	668	NSS	HexNAc(4)Hex(5)Fuc(1)
14	688	NVT	HexNAc(2)Hex(6)	HexNAc(2)Hex(7)	HexNAc(2)Hex(6)	689	NVT	HexNAc(2)Hex(7)
15						723	NST	HexNAc(6)Hex(7)Fuc(1)[c]
16	726	NST				727	NST	
17	743	NCT	HexNAc(4)Hex(5)Fuc(1)NeuAc(1)	HexNAc(4)Hex(5)Fuc(1)NeuAc(1)	HexNAc(4)Hex(5)Fuc(1)NeuAc(1)	744	NCT	HexNAc(6)Hex(7)Fuc(1)
18	781	NIS				782	NIS	
19	787	NFS				788	NFS	
20	873	NFT	HexNAc(2)Hex(7)[b]	HexNAc(2)Hex(5)	HexNAc(2)Hex(5)	874	NFT	HexNAc(2)Hex(5)
21	1009	NIT	HexNAc(2)Hex(8)	HexNAc(2)Hex(8)	HexNAc(2)Hex(9)	1010	NIT	HexNAc(2)Hex(8)
22[a]	1196	NHT	HexNAc(4)Hex(6)Fuc(1)NeuAc(1)	HexNAc(6)Hex(7)Fuc(1)[c]	HexNAc(6)Hex(3)Fuc(1)	1197	NYT	HexNAc(6)Hex(7)Fuc(1)NeuAc(2)
23	1232	NLT			HexNAc(4)Hex(3)Fuc(1)[c]	1233	NLT	
24	1249	NKT	HexNAc(4)Hex(5)	HexNAc(4)Hex(5)	HexNAc(6)Hex(4)Fuc(1)[c]	1250	NKT	HexNAc(6)Hex(7)
25	1261	NRT	HexNAc(5)Hex(6)Fuc(1)	HexNAc(4)Hex(5)Fuc(1)	HexNAc(6)Hex(3)Fuc(1)	1262	NRT	HexNAc(6)Hex(7)Fuc(1)NeuAc(1)[c]
26	1273	NAT				1274	NAT	
27	1278	NLT	HexNAc(2)Hex(5)		HexNAc(6)Hex(3)	1279	NLT	
28	1295	NTT			HexNAc(6)Hex(7)Fuc(1)NeuAc(2)	1296	NTT	HexNAc(6)Hex(7)Fuc(1)NeuAc(1)
29	1308	NNT			HexNAc(6)Hex(6)Fuc(1)NeuAc(2)[b]	1309	NNT	HexNAc(6)Hex(7)Fuc(1)NeuAc(3)

The glycopeptides were identified by both Byonic and pGlyco3 and quantified by Byos and pGlyco3 based on the area under the curve of their respective extracted ion chromatograms. The glycosyl compositions listed here correspond to the glycoforms with the highest peak areas for each site that were used in structural modeling of the intact glycoprotein. Cells shaded in gray represent sites for which the non-glycosylated form actually afforded the highest peak area.

a. Synonymous but not identical sequons

b. The glycoform is identified by pGlyco3 only

c. The glycoform is identified by Byonic only

Supplementary Table 2. Sequence identity of different PEDV strains

Sequence identity (%)			G2 PEDV	
			CO/13 [b]	PT52 [c]
G1 PEDV	CV777[a]	S1+S2	93.7	92.7
		S1	91.0	90.2
		S2	96.9	96.5
G2 PEDV	CO/13 [b]	S1+S2		99.1
		S1		99.0
		S2		99.2

a. The sequence is based on Genebank entry NP_598310.1. The S1 and S2 sequences correspond residues 34-761 and 762-1242, respectively.
b. The sequence is based on Genebank entry AGO58924.1. The S1 and S2 sequences correspond residues 34-764 and 765-1245, respectively.
c. The sequence is based on Genebank entry ART85704.1. The S1 and S2 sequences correspond residues 34-764 and 765-1245, respectively.

Supplementary Table 3. Summary of cryo-ET Data acquisition and image processing

	PEDV S D0$_{DDD}$	PEDV S D0$_{UDD}$	PEDV S D0$_{UUD}$	PEDV S D0$_{UUU}$	PEDV S postfusion
Data Collection					
Microscope			FEI Titan Krios		
Voltage (kV)			300		
Detector			Gatan K3 Summit		
Recording Mode			Counting		
Pixel Size (Å)			5.558 (bin 4)		
Defocus Range (μm)			1.5 to 6		
Acquisition Scheme			Bidirectional, 20° to -60° then 23° to 60°, with 3° steps		
Total Dose ($e^-/Å^2$)			~100 or ~120		
Frame number			9 or 10		
Tomograms			91		
Image Processing					
Viral Particles			462		
Subtomograms	2385	2747	1355	211	132
Symmetry imposed	C3	C1	C1	C3	C3
Map resolution (Å) FSC threshold 0.143	19	27	25	29	31
EMDB ID	EMD-32332	EMD-32333	EMD-32337	EMD-32339	EMD-32340

Supplementary Table 4. Summary of the cryo-EM data collection parameters and model statistics of intact viral particles.

	PEDV S $D0_{DDD}$ (EMD-32329) (PDB 7W6M)	PEDV S $D0_{UDD}$ (EMD-32338) (PDB 7W73)
Data collection and processing		
Magnification	×64 000	
Voltage (kV)	300	
Electron exposure (e^{-}/Å^2)	55.4	
Defocus range (μm)	1 to 3	
Pixel size (Å)	1.4	
Symmetry imposed	C3	C1
Initial particle images (no.)	337 583	
Final particle images (no.)	19 350	9 319
Map resolution (Å)	4.7	6.4
FSC threshold	0.143	0.143
Refinement		
Initial model used (PDB code)	Swiss-Model homology mode based on 6VV5 template	
Model resolution (Å)	4.7	6.5
FSC threshold	0.143	0.143
Map sharpening *B* factor (Å^2)	-184.6	-331.4
Model composition		
Non-hydrogen atoms	30759	30 899
Protein residues	3672	3672
Ligands	MAN:45	MAN:59
	BMA: 39	BMA: 43
	NAG: 108	NAG: 106
	FUC:12	FUC:9
B factors (Å^2)		
Protein	90.00	374.17
Ligand	158.56	465.00
R.m.s. deviations		
Bond lengths (Å)	0.003	0.004
Bond angles (°)	0.596	0.744
Validation		
MolProbity score	1.79	2.09
Clashscore	9.13	12.79
Poor rotamers (%)	0	0
Ramachandran plot		
Favored (%)	95.58	92.42
Allowed (%)	4.42	7.45
Disallowed (%)	0	0.14

Supplementary Table 5. Summary of the cryo-EM data collection parameters and model statistics of recombinant PEDV PT52 S from IPEC-J2.

	PEDV S $D0_{UUU}$ (EMD-33646) (PDB 7Y6S)	PEDV S $D0_{UUD}$ (EMD-33647) (PDB 7Y6T)	PEDV S single protomer D0-up with CTD-close (EMD-33648) (PDB 7Y6U)	PEDV S single protomer D0-up with CTD-open (EMD-33649) (PDB 7Y6V)
Data collection and processing				
Magnification	×81 000			
Voltage (kV)	300			
Electron exposure ($e^{-}/Å^2$)	50			
Defocus range (μm)	1.8 to 2.3			
Pixel size (Å)	1.06			
Symmetry imposed	C1	C1	C1	C1
Initial particle images (no.)	396 287		894 585	
Final particle images (no.)	298 195	51 124	171 864	124 142
Map resolution (Å)	3.1	4.2	3.2	3.3
FSC threshold	0.143	0.143	0.143	0.143
Refinement				
Initial model used (PDB code)	7W6M in this study			
Model resolution (Å)	3.1	4.1	3.3	3.4
FSC threshold	0.143	0.143	0.143	0.143
Map sharpening *B* factor ($Å^2$)	98.6	78.5	94.8	104.1
Model composition				
Non-hydrogen atoms	24855	27991	9916	9989
Protein residues	3012	3446	1224	1224
Ligands	MAN:12<br>BMA: 18<br>NAG: 81<br>FUC:12	MAN:6<br>BMA: 18<br>NAG: 82<br>FUC:9	MAN:2<br>BMA: 7<br>NAG: 27<br>FUC:4	MAN:2<br>BMA: 6<br>NAG: 33<br>FUC:4
B factors ($Å^2$)				
Protein	154.57	727.89	133.11	146.03
Ligand	223.61	761.26	174.61	194.17
R.m.s. deviations				
Bond lengths (Å)	0.003	0.007	0.003	0.003
Bond angles (°)	0.598	0.880	0.533	0.604
Validation				
MolProbity score	1.81	2.62	1.80	1.92
Clashscore	9.26	40.32	7.40	9.90
Poor rotamers (%)	0.23	0.41	0	0
Ramachandran plot				
Favored (%)	95.50	90.38	94.19	94.03
Allowed (%)	4.33	9.18	5.48	5.73
Disallowed (%)	0.17	0.44	0.33	0.25

Supplementary Table 6. Summary of the cryo-EM data collection parameters and map statistics of recombinant PEDV PT52 S from HEK293F.

	PEDV PT52 S WT			PEDV PT52 S T326I			
	$D0_{DDD}$	$D0_{UUD}$	$D0_{UUU}$	$D0_{DDD}$	$D0_{UDD}$	$D0_{UUD}$	$D0_{UUU}$
Data Collection and processing							
Magnification				×81 000			
Voltage (kV)				300			
Electron exposure ($e^{-}/Å^2$)				50			
Defocus Range (μm)				1.8 to 2.3			
Pixel Size (Å)				2.2			
Symmetry imposed	C3	C1	C1	C1	C1	C1	C1
Initial particle images (no.)		95 371			99 985		
Final particle images (no.)	10 676	6 446	78 249	42 239	15 682	19 273	22 791
Map resolution (Å)	5.6	10.2	4.5	5.2	8.0	6.1	6.2
FSC threshold	0.143	0.143	0.143	0.143	0.143	0.143	0.143
EMDB ID	EMD-33700	EMD-33701	EMD-33702	EMD-33703	EMD-33704	EMD-33705	EMD-33706
