# Supplementary material for: In situ structure and dynamics of an alphacoronavirus spike protein by cryo-ET and cryo-EM
Source: Nat Commun. 2022 Aug 19;13:4877. doi: 10.1038/s41467-022-32588-3 (PMC9388967; doi:10.1038/s41467-022-32588-3)
Supplement: Supplementary file 3 — Description of Additional Supplementary Files [file 41467_2022_32588_MOESM3_ESM.pdf]

Description of Additional Supplementary Files

File Name: Supplementary Movie 1
Description: In situ cryo-electron tomogram of PEDV

File Name: Supplementary Movie 2
Description: The stalk region of the PEDV prefusion S protein has flexibility which allows the spike to bend over freely with respect to the viral membrane.
