# Supplementary material for: In situ structure and dynamics of an alphacoronavirus spike protein by cryo-ET and cryo-EM
Source: Nat Commun. 2022 Aug 19;13:4877. doi: 10.1038/s41467-022-32588-3 (PMC9388967; doi:10.1038/s41467-022-32588-3)
Supplement: Supplementary file 6 — Reporting Summary [file 41467_2022_32588_MOESM6_ESM.pdf]

nature portfolio

Corresponding author(s): Shang-Te Danny Hsu, NCOMMS-21-48954A

Last updated by author(s): Aug 3, 2022

Reporting Summary

Nature Portfolio wishes to improve the reproducibility of the work that we publish. This form provides structure for consistency and transparency in reporting. For further information on Nature Portfolio policies, see our Editorial Policies and the Editorial Policy Checklist.

Statistics

For all statistical analyses, confirm that the following items are present in the figure legend, table legend, main text, or Methods section.

n/a	Confirmed	
☐	☒	The exact sample size (*n*) for each experimental group/condition, given as a discrete number and unit of measurement
☒	☐	A statement on whether measurements were taken from distinct samples or whether the same sample was measured repeatedly
☒	☐	The statistical test(s) used AND whether they are one- or two-sided<br>*Only common tests should be described solely by name; describe more complex techniques in the Methods section.*
☒	☐	A description of all covariates tested
☒	☐	A description of any assumptions or corrections, such as tests of normality and adjustment for multiple comparisons
☐	☒	A full description of the statistical parameters including central tendency (e.g. means) or other basic estimates (e.g. regression coefficient) AND variation (e.g. standard deviation) or associated estimates of uncertainty (e.g. confidence intervals)
☒	☐	For null hypothesis testing, the test statistic (e.g. *F*, *t*, *r*) with confidence intervals, effect sizes, degrees of freedom and *P* value noted<br>*Give P values as exact values whenever suitable.*
☒	☐	For Bayesian analysis, information on the choice of priors and Markov chain Monte Carlo settings
☒	☐	For hierarchical and complex designs, identification of the appropriate level for tests and full reporting of outcomes
☒	☐	Estimates of effect sizes (e.g. Cohen's *d*, Pearson's *r*), indicating how they were calculated

Our web collection on statistics for biologists contains articles on many of the points above.

Software and code

Policy information about availability of computer code

Data collection	EPU 2.0, Tomography 4.0
Data analysis	IMOD 4.11.6, ETOMO, EMAN2, Chimera 1.13, PlaceObject 2.0, ChimeraX 1.2.5, ISOLDE, MATLAB R2020a, Dynamo 1.1.514, Swiss-MODEL, MotionCor2, CTFFIND 4.1, Relion 3.1, cryoSPARC 3.1, Coot 0.9.6, PHENIX 1.20.1-4487, Byonic 3.9.6, Byos with Byologic 4.053, pGlyco 3.0<br>Custom code for our cryoET data analysis: https://github.com/Koushouu/initial_template_generation

For manuscripts utilizing custom algorithms or software that are central to the research but not yet described in published literature, software must be made available to editors and reviewers. We strongly encourage code deposition in a community repository (e.g. GitHub). See the Nature Portfolio guidelines for submitting code & software for further information.

Data

Policy information about availability of data

All manuscripts must include a data availability statement. This statement should provide the following information, where applicable:

- Accession codes, unique identifiers, or web links for publicly available datasets
- A description of any restrictions on data availability
- For clinical datasets or third party data, please ensure that the statement adheres to our policy

Protein Data Bank (PDB) entries: 7W6M, 7W73, 7Y6S, 7Y6U, 7Y6V
Electron Microscopy Data Bank (EMDB) entries: EMD-32329, EMD-32338, EMD-33646, EMD-33647, EMD-33648, EMD-33649, EMD-33702, EMD-33701,

EMD-33700, EMD-33706, EMD-33705, EMD-33704, EMD-33703, EMD-32332, EMD-32333, EMD-32337, EMD-32339, EMD-32340
Mass Spectrometry Interactive Virtual Environment (MassIVE) Database entry: MSV000088544

Human research participants

Policy information about studies involving human research participants and Sex and Gender in Research.

Reporting on sex and gender	N/A
Population characteristics	N/A
Recruitment	N/A
Ethics oversight	N/A

Note that full information on the approval of the study protocol must also be provided in the manuscript.

Field-specific reporting

Please select the one below that is the best fit for your research. If you are not sure, read the appropriate sections before making your selection.

- [x] Life sciences
- [ ] Behavioural & social sciences
- [ ] Ecological, evolutionary & environmental sciences

For a reference copy of the document with all sections, see nature.com/documents/nr-reporting-summary-flat.pdf

Life sciences study design

All studies must disclose on these points even when the disclosure is negative.

Sample size	The sample size was determined by the available electron microscopy time and the amount of virus sample. The sample size was sufficient to obtain a structure at the resolution stated in the manuscript, to obtain a biological conclusion. The mass spectrometry glycoform analyses were carried out using single samples, which could be produced reliably using the protocols defined in the Methods section.
Data exclusions	Electron microscopy images that could not be used due to ice contamination, blurring due to the error during imaging, or images that are hindered by the grid bars are excluded during the data analysis. Glycopeptide analyses were carried out following the selection criterion defined in the Methods section to ensure statistical significance.
Replication	For both the cryoEM and cryoET image processing pipelines the data were divided to independent half set at the final stage for calculation of resolutions.
Randomization	During the subtomogram classification in the cryoET data analysis a volume with random noise was introduced to rule out the false positives in the dataset
Blinding	Blinding was not applicable to this study as group allocation was not required in this type of study

Reporting for specific materials, systems and methods

We require information from authors about some types of materials, experimental systems and methods used in many studies. Here, indicate whether each material, system or method listed is relevant to your study. If you are not sure if a list item applies to your research, read the appropriate section before selecting a response.

Materials & experimental systems

n/a	Involved in the study	
x		Antibodies
	x	Eukaryotic cell lines
x		Palaeontology and archaeology
x		Animals and other organisms
x		Clinical data
x		Dual use research of concern

Methods

n/a	Involved in the study	
x		ChIP-seq
x		Flow cytometry
x		MRI-based neuroimaging

Eukaryotic cell lines

Policy information about cell lines and Sex and Gender in Research

Cell line source(s)	Vero C1008 cells (ATCC No. CRL-1586)<br>HEK293 cells (ATCC No. CRL-1573)<br>Intestinal porcine epithelial cell line-J2 (IPEC-12) cells (ACC701; Leibniz Institute DSMZ - German Collection of Microorganisms and Cell Cultures, Braunschweig, Germany)<br>FreeStyleTM 293-F Cells（R79007, Gibco）
Authentication	None of the cell line used were authentiated
Mycoplasma contamination	Cell lines were tested negative for mycoplasma contamination by the providers
Commonly misidentified lines (See ICLAC register)	No commonly misidentified cell lines were used in the study
